# Household survey of availability of long-lasting insecticide-treated nets and its determinants in rural Mozambique

**DOI:** 10.1186/s12936-015-0811-3

**Published:** 2015-08-06

**Authors:** Inocencio M Quive, Baltazar Candrinho, Diederike Geelhoed

**Affiliations:** Tete Provincial Health Directorate, Tete City, Mozambique; Danish Ministry of Foreign Affairs, Provincial Directorate of Health, Tete City, Tete Province Mozambique

**Keywords:** Long-lasting insecticide-treated nets, Malaria prevention, Mozambique

## Abstract

**Background:**

Mosquito nets treated with long-lasting insecticide offer highly effective personal protection against malaria transmission. In Mozambique, nets are distributed freely in antenatal care visits since 2006 and through mass distribution campaigns since 2009, but the country has not yet been able to report a consistent decline in malaria incidence. Routine data show that Changara District, Tete Province, shows an increase in malaria cases, although it has a reasonable theoretical coverage of nets. This study evaluated household availability of nets and its determinants in Changara district.

**Methodology:**

Quantitative household survey at the end of 2013, in a representative sample of 450 households in 30 villages of Changara district, using the sampling method of randomly selected households in clusters selected with probability proportional to size. Data were analysed with Epi-Info version 7.1.2.0. The significance level was 0. 05.

**Results:**

Of 450 households, 62.5% (95% CI 57.5–66.7) had at least one long-lasting insecticide-treated net. Availability of nets showed a positive association with socioeconomic status and the existence of at least one pregnant woman or child under 5 years in the household, but a negative association with distance between health facility and residence. Most of the observed nets were not in good condition, only 19.2% (95% CI 15.7–23.2) of households had at least one net in good condition. The condition of the nets reduced with increasing number of washes.

**Conclusions:**

The household availability of long-lasting insecticide-treated nets in Changara district has not yet reached levels that may have an impact on the incidence of malaria, despite distribution through campaign and antenatal care. The habit of washing nets frequently reduces their lifespan. It is recommended to strengthen education on good practices of net conservation, in addition to their distribution.

## Background

Consistent and correct use of long-lasting insecticide-treated nets (LLITN) does reduce malaria incidence in under fives by up to 50%, incidence of severe malaria by 45%, and infant mortality by 25% [1–4]. Universal coverage of LLITNs is defined as the use of a net by more than 80% of the population at risk, counting one net for each two persons [5]. Consensus in the malaria Vector Control Working Group (VCWG) indicates that universal coverage, reached through mass distribution campaigns, is the main strategy for the rapid attainment of malaria control [4]. Mozambique, a malaria endemic country, has introduced mass distribution campaigns every 3–4 years in selected districts since 2009, in addition to distribution through antenatal care. However, the country is not yet able to report a consistent decline in malaria incidence [6, 7].

The physical integrity of a net is important for its level of protection against malaria [8]. Studies on the conservation of nets suggest that considerable damage frequently occurs already within 1 year, related to poor maintenance. This damage increases with duration of use. Many nets do not last for more than 2 years, although some have stated that lifespan of nets can be up to 4 years [1, 2, 9–12].

Tete Province, in central Mozambique, is one of the areas showing a recent increase in malaria incidence, despite the distribution of a considerable number of nets since 2010. Changara is the district in Tete Province which, theoretically, based on net distribution numbers relative to the population, should have the highest net coverage in the province, close to universal, assuming an effective lifespan of around 3–4 years. Nevertheless, although malaria incidence per 1,000 population was 34 in 2010 (when the mass distribution was done), routine data show an increasing incidence of malaria since then, to 64 in 2013. If the actual net coverage in the district would be lower than expected, or the physical integrity of the nets compromised, this might explain the apparent contradiction between expected net coverage and reported malaria incidence.

This study evaluated coverage and conservation status of LLITN in Changara district, to shed light on the apparent contradiction between expected net coverage and reported malaria incidence.

## Methods

A household survey was implemented in Changara district, Tete province, Mozambique, with data collection in December 2013, during the rainy season. The district was purposely selected, based on its apparent contradiction between expected net coverage and reported malaria incidence. The public health system in Changara district consists of 13 outpatient units and one inpatient unit serving a population of 189,000 [13], indicating a low ratio of health care facilities per inhabitant (1/13,400). The only mass LLITN distribution campaign in the district was in 2011, but distribution through antenatal care is on-going. The district has a semi-arid climate with strong seasonal differences in rainfall, and its inhabitants are mainly subsistence farmers [14]. A modified cluster sample survey (30 × 15) was employed [15]. The sample was obtained in two steps. First, 30 conglomerates were randomly selected, proportional to their population size. Then, 15 households per conglomerate were randomly selected (systematically), totaling 450 households in the sample. The questionnaire, applied to one representative from each household, was based on previous questionnaires of similar surveys in Mozambique.

The study variables were classified in two categories:

*Socio*-*demographic details* Age, gender and educational level of household representative, number of pregnant women and children under 5 years of age in household, total number of people in the household, construction material of the walls and roof of the household house, and household ownership of assets (livestock);

*Characteristics related to LLITN* Presence of nets in the household, source of each net (free distribution through campaign or prenatal consultation by non-governmental organizations, commercial purchase), time passed since reception of each net (net age), laundry habits of nets (number of times washed), physical integrity of each net (number of holes with a diameter greater than 1 cm).

The questionnaire was applied by one of five trained research assistants, under supervision of two principal investigators, with support from a community representative in each conglomerate, who assisted with translation from Portuguese into the local language. Quality of data collection was ensured during daily meetings of the entire team during the field work.

The statistical package EPI-Info v 7.1.2.0 was used to digitalize, verify, and analyse data. Frequencies were calculated for all variables with their confidence intervals. Educational level was combined with house construction material and ownership of assets into a new variable indicating the socioeconomic status of each household. Chi square tests were used to detect statistically significant differences between groups, with a significance level of 0.05.

The study was implemented within routine activities of the provincial malaria control programme, with authorization from the Provincial Health Authorities, District Government and local community leaders. Written informed consent was obtained from each participating household representative.

## Results

During data collection, 30 villages were visited and 450 household representatives interviewed, 15 households per village. No household was excluded, and no representatives denied participation. The socio-demographic characteristics of the participants are summarized in Table 1.

**Table 1:** Socio-demographic characteristics of the participants

Variable	Categories	N	%	95% CI
Gender	Female	287	63.8	59.1–68.2
	Male	163	36.2	31.8–40.9
Representative	Household head	223	49.6	44.9–54.3
	Other	227	50.4	45.7–55.2
Socio-economic level	Lowest 25%	164	38.9	34.2–43.7
	Medium–low 25%	143	33.9	29.4–38.7
	Medium–high 25%	100	23.7	19.8–28.1
	High 25%	15	3.6	2.1–5.9
Distance to nearest health facility	Close to health facility <10 km	225	50.0	45.2–54.8
	Far from health facility >10 km	225	50.0	45.2–54.8
Household members	Total number in all households	2,612	100	–
	Mean number per household	5.8 (SD 3.0); range 1–18		
	Number of households with at least one pregnant women	37	8.2	5.9–11.3
	Number of households with at least one under five	300	66.7	62.1–71.0

Results regarding household availability and physical integrity of LLITN are summarized in Table 2. Among the participating households, 62.5% were found to have at least one LLITN, but only 19.2% of households were found to have at least one LLITN with good physical integrity. The mean number of persons per household was 5.8 (SD 3.0), however, the mean number of LLITN in any condition per household was only 1. 1 (SD 1.2), and the mean number of LLITN with good physical integrity was just 0.3 (SD 0.6). Even in households with at least one LLITN in good condition, the mean number of LLITN in good condition was low (1.4, SD 0.7) compared to the mean number of persons (6.5, SD 2.9). Overall, most of the nets (75.5%; 95% CI 69.5–80.0) originated from the 2011 mass distribution campaign, followed by antenatal care distribution (16.5%; 95% CI 10.7–24.0).

**Table 2:** Household availability LLITN

Variable	Categories	N	%	95% CI
Availability of LLITN	Number of households with at least one LLITN (regardless of its condition)	280	62.5	57.8–67.0
	Number of households without any LLITN (regardless of condition)	168	37.5	33.0–42.2
	Number of households with at least one LLITN (in good condition)	86	19.2	15.7–23.2
	Number of households without any LLITN (in good condition)	362	80.8	76.8–84.3

Determinants of household availability of at least one LLITN (regardless of its condition) included socioeconomic status, where households with a lower status were less likely to have at least one LLITN, than those with a higher status (OR 0.6; 95% CI 0.4–0.9; p = 0.02). Distance to the nearest health facility also determined household LLITN availability: those living more than 10 km from a health facility were less likely to have at least one LLITN, than households who lived closer (OR 0.6, 95% CI 0.4–0.9; p = 0.006). In addition, the existence of a pregnant woman or under five was associated with household LLITN availability: households with at least one pregnant woman or under five were more likely to have at least one LLITN than households without pregnant woman or under five (OR 1.5; 95% CI 1.0–2.2; p = 0.03).

In those households with at least one LLITN, availability of at least one net with good physical integrity was associated again with socioeconomic status, where households with a lower status were less likely to have at least one LLITN, than those with a higher status (OR 0.5; 95% CI 0.3–0.9; p = 0.08). It was also determined by area of residence: households living in the least accessible part of the district were less likely to have at least one good net, than households who lived elsewhere (OR 0.5, 95% CI 0.3–0.8; p = 0.004). In addition, it was associated with the existence of pregnant women or under fives in the household: households with at least one pregnant woman or under five were more likely to have at least one good LLITN than households without pregnant woman under five (OR 1.8; 95% CI 1.0–3.3; p = 0.02). Finally, there was an association with washing practices: those households which already had washed their LLITN four times or more were less likely to have at least one LLITN in good condition that the households which had washed less often (OR 0.5; 95% CI 0.3–0.8; p = 0.002).

## Discussion

This survey in Changara District raises important issues for local malaria control with LLITN. The WHO and the Roll-Back Malaria Initiative recommend 100% universal coverage with LLITN to long-term high-risk populations (counting one net for each two persons) with a target of at least 80% utilization [5]. The results showed a high percentage of households without any LLITN, and an insufficient mean number of LLITN with good physical integrity as compared to the mean number of persons per household. Even in households which had at least one LLITN with physical integrity, the mean number of LLITN was inadequate to protect all household members. Therefore, the current LLITN availability in Changara is far from ideal, which probably explains the high malaria incidence locally.

The study results suggest that low LLITN availability is related to the limited effective lifespan of the LLITN, mainly due to inappropriate washing practices. Instead of a potential lifespan of up to 4 years, the local LLITN seem to keep their integrity during fewer years, as reported in others studies [16, 17]. Poor maintenance of nets and inappropriate washing practices have also been reported by other studies, which suggest that the usefulness of the net decreases rapidly through physical damage and reduction of the effect of the insecticide related to washing, amongst other factors [18, 19]. This study suggests the need to extend the lifetime of the LLITN through education programmes in the community on appropriate ways of handling, washing and repair, which has been shown feasible and effective elsewhere [20].

The results show that many LLITN were provided through universal distribution campaign, and relatively few through distribution in antenatal care, although households with pregnant women or under fives were more likely to possess a LLITN. This indicates an insufficient capacity of antenatal care distribution to provide initial LLITN and their timely replacement, once no longer in good condition, also observed in other studies [21]. This suggests the need to strengthen the distribution channels, with more frequent campaigns to rapidly increase coverage, and increased distribution through health facilities to maintain elevated rates [22]. As a distribution channel, the universal distribution campaign is highly effective and should be preferred in view of the inequities observed in this study with regard to socio-economic status and distance to a health facility, despite its major logistical burden [23]. The most disadvantaged groups in a population access health care less easily, and therefore distribution by health facility is inherently inequitable.

There are some limitations to our study. It was difficult to measure consistently and rigorously the physical integrity of the LLITN in the field. Insecticide content of the nets was also not measured. Therefore only LLITN without any visible holes (>1 cm) were considered as in good condition, which may have been excessively strict. However, there is some evidence that mosquitoes indeed can enter in torn nets [16]. No information on malaria incidence in the study population was gathered, and another limitation is the lack of information on bed net use. However, as so few households had at least one LLITN in good condition, even with optimal usage the protection offered in the population would be limited.

## Conclusions

The LLITN household availability in Changara district has not yet reached levels that may have an impact on the incidence of malaria, despite distribution through campaign and antenatal care. The lifespan of the LLITN appears to be much shorter than previously assumed, related to inappropriate washing practices. Some level of inequity in LLITN availability was observed associated with distance to health facility, socioeconomic status, and residence in less accessible areas, although households with pregnant women or under fives were more likely to possess a LLITN. Our study suggests a need to increase the frequency of mass distribution campaigns and strengthening routine distribution through health facilities, intensifying education programmes on net maintenance to reduce the LLITN attrition rate, in order to increase household availability and consequently reduce the burden of malaria in rural Mozambique.
